# Enhancing Group Bonding in an Acting Class of Older Adults in Urban Subsidized Housing

**DOI:** 10.1093/geroni/igab046.3319

**Published:** 2021-12-17

**Authors:** Laura Sutherland, Ruth Dunkle, Garrett Pace

**Affiliations:** 1 Wayne State University, Ann Arbor, Michigan, United States; 2 University of Michigan, Ann Arbor, Michigan, United States

## Abstract

Creative arts such as acting can promote social contact and bonding among socially isolated populations. Yet the benefits of art programming among older adults in low-income urban settings remain unexplored. A professionally administered theater group comprised of older adults living in urban low-income housing met for 12 weeks to learn acting skills and perform a play. The purpose of this study was to identify: 1) why participants enroll in a residence-based acting and improvisation course, and 2) what aspects of the course contribute to group bonding. Participants (n=14) were African American. The average age was 63 years, 14% were men, 57% had a high school degree or less, 79% reported good to excellent health, and the mean ADL score was 1.45 (range: 1-2.5). A researcher was present at each class session to observe and take field notes. Pre-post interviews included closed and open-ended questions. The researchers reviewed field notes and interview transcripts for a priori themes and emergent themes through independently coding data, discussing similarities and discrepancies, and coming to consensus on themes. Results showed that participants were motivated to enroll to meet new people, come out of themselves, learn something new, and gain artistic skills. Participants indicated the course contributed to group bonding via teaching techniques, course structure, the teacher’s use of self, the expression of self, and mutually enhanced coping skills. Results from this study provide guidance for the design of theater groups in similar settings and inform recruitment efforts of older adults in creative arts programs.

